# The Art of Speech: Elocution, Speech Training, Speech Therapy, and the Performative Limits of Class in Mid-twentieth-century Britain

**DOI:** 10.1093/tcbh/hwae043

**Published:** 2024-05-15

**Authors:** Andrew Burchell

**Affiliations:** University of Warwick

## Abstract

This article argues that elocution, speech training, and speech therapy—three professions concerned with the voice—were intimately bound up with a shifting politics of class in early- and mid-twentieth-century Britain. The last two, in particular, attempted to stake new claims in the changed landscape of the post-1945 welfare state. Proponents of speech training distinguished themselves from elocutionists and saw their role to improve children’s speech, but they performatively disavowed class as an organizing category within it. This was paralleled by speech therapy, which emerged as a formal profession in Britain in 1945 through the unification of two separate (and often rival) halves of the profession under a single regulatory college, and which found itself having to justify where its pathologizing of vocal production ended and elocution’s focus on the aesthetics of accent began. I argue that these disavowals provide a useful framework through which to read class dynamics and consider the performative dimensions of class identities at this time. Mobilising select writers and speech experts—who straddled the boundaries of elocution, speech training, and speech therapy—this article shows how a variety of different categories, from gender to geography, were employed as proxies to allow for the problematization of dialect but not accent and to efface ‘class’.

In the mid-1930s, a series of advertisements appeared in the English provincial press—placed by ‘Mr S. Drayton Raw’, ‘the London Speech Specialist’—publicizing a national lecture tour.^1^ Intrigued attendees could equally seek a brochure for Raw’s ‘British Institute of Speech’, from whose conveniently-located studio in central London (and in exchange for fees dependent on the ‘severity of the case’ and ‘the amount of personal attention necessary’) Raw offered bespoke, individualized treatment for people who stammered.^2^ But there is more to Raw’s claims in this grey literature—whether we choose to view them charitably as humanitarian or, less positively, as mere quackery—than meets the eye. The address given for his ‘British Institute of Speech’ sites its headquarters on Marylebone High Street. Such prime London real estate offered him a geographical location close to both the Royal Academy of Music (an institution which, throughout the inter-war years, produced a large number of elocutionists who would later enter the discipline of speech therapy) and Harley Street, with claims to a very different (and more securely scientific) form of medical authority. The choice of this location was almost certainly not an accidental one on Raw’s part, and he may have hoped that his commercial venture would bask in the reflected glories of both visions of speech: the artistic world of vocal performance and scientific medicine’s focus on normativity and rehabilitation.

We might contextualize Raw’s crossings of medical and artistic boundaries with reference to other examples of passing between the world of medical expertise and aesthetics.^3^ Indeed, there was a varied ‘medical marketplace’ (to employ Roy Porter’s term)^4^ at the beginning of the twentieth century for the ‘cure’ of ‘speech defects’ and the improvement of the spoken voice more generally.^5^ Yet, as Raw’s pledge to instil ‘well-articulated English free from foreign or provincial accents’ alongside treatment for stammering suggests,^6^ there is equally an important story here about class, identity and voice in the middle years of the twentieth century. Perhaps most revealing, in this regard, was Raw’s chosen area of ‘disordered’ speech: stammering. This, he asserted grandly, and with a possible nod to George Windsor’s stammer, ‘makes no distinction of classes […] From the university graduate to the humble artisan, from the nobleman to the labourer, from rich and poor alike, stammering takes an equal toll’.^7^ Even if his high fees implicitly favoured those most able to pay and belied such grandiose declarations of solidarity, it is rather the desire to appeal across class boundaries which matters here.

The early- and mid-twentieth century witnessed developments in several interconnected movements and professions concerned with the voice and its alleged faults. The first of these, ‘elocution’, had been inherited from the nineteenth century and was categorized by a set of vocal and performative practices, typified by recitations. It progressively became institutionalized in the first decades of the twentieth century. Secondly, there was a partial shift away from ‘elocution’ towards ‘speech training’, which drew from more consciously medical and anatomical discourses to legitimate and promote the aims of ‘good’ and clear speech. Lastly, ‘speech therapy’ emerged as a medical specialism dealing with speech explicitly classified as in some way pathological, like Raw’s stammering cases. In considering these three movements and their key protagonists together, as mutually implicated and not just parallel developments, this article argues for important continuities in how these groups approached class and accent. The disavowal of class made by Raw was, to a greater or lesser extent, found across elocution, speech training, and speech therapy texts. The tensions and contradictions produced by the proponents of these three specialisms allow us to read transitions in how voice professionals understood the relationship between voice and class in Britain, and to explore how issues of gender and geography increasingly acted as proxies for concern around the ‘classed’ voice. I argue that the period immediately before and after the Second World War was marked by a shifting discourse within ‘speech training’, implicating elocutionists and speech therapists on common ground. This was typified by porous boundaries between ‘medicalized’ and ‘standardized’ or ‘normative’ interventions in speech. The kinds of performative ‘passing’ this enabled therefore included movements across different professional boundaries as much as between classes.

This article begins with an overview of interactions between the voice and class in the historiography of modern Britain, before moving onto a discussion of elocution, speech training, and speech therapy—further examining their connections and exploring their institutionalization and standardization from the beginning of the twentieth century to the post-war period. The main section then examines published output from members of the three professional groups who wrote about accent, voice and class between the 1930s and 1950s. These will demonstrate some level of convergence—but also key variations—in dealing with accent and dialect as markers of class at this welfare state moment. Finally, the article traces the decline of the two artistic and aesthetic endeavours (speech training and elocution), which faded at exactly the moment that speech therapy succeeded in becoming medicalized.

## Class, ‘Performative’ Speaking and British History

The key selling point of figures like Raw was that changes in the voice were aspirational. Raw’s advertising copy played on anxieties around social and cultural capital as much as medicalized concerns around stammering. Removing the ‘pathology’ allowed space to rehabilitate the voice on standardized lines that did not betray geographical or socio-economic origin. In virtually all the elocution, speech training, and speech therapy work at this time, the voice was inherently and overtly considered as ‘performative’ in a literal, and not just a theoretical, sense.^8^ The voice, speech trainer and therapist Anne McAllister informed her child readers, was a versatile ‘musical instrument, the most perfect in the world’; while proponents of elocution and speech training offered guidance to how the voice could express poetic metre and feeling.^9^ If we accept the basic premise of its practitioners—that speech^10^ and aspects of vocal tone and accent are essentially malleable and can thus be trained to sound different from their original state—then we can see these practices as enablers of a radical act of cross-class passing rather than uniquely as conservative forces preserving hierarchies of accent. Part of the appeal of speech training and speech therapy from the immediate pre-war to post-war period is that they appeared (at least for a short moment) to contain these possibilities and the potential both for ‘passing’ and ‘abjection’.

This latter term derives from Judith Butler’s characterization of sites in which the performativity of gender is simultaneously most visible and therefore most threatening to those seeking to impose difference as essentialized and hence normative. At the same time, Butler argues that ‘abjection’ can be read as emphasizing the inherent ‘instability’ and liminality of identities.^11^ The life narrative of Hilda Wooler (b.1903), an Accrington-based elocution teacher for a large part of the early and mid-twentieth century, betrays something of these processes. There is, in the first instance, her own Lancashire accent in the recorded oral history interview with her (undertaken by the local historian, Benita Moore, in 1993). The extent and strength of Wooler’s accent varies; it is quite light when she actually gives a poetry recitation to demonstrate her elocution skills, compared to the substance of the discussion in the interview, suggesting the curation on Wooler’s part of a performative mode or character. Secondly, while often being guarded about her own class background, Wooler does allude to her mother’s shocked reaction to her daughter’s insistence on learning elocution, telling the young Hilda that lessons were ‘going to cost a lot of money’. Wooler subsequently contrasts her class background quite overtly to that of her elocution teacher, ‘Mrs R.A. Waddington at Blackburn’, with whom she studied from the age of fourteen to twenty-one.^12^ Waddington was, she recalls, ‘a real, what you might call lady. Most of her pupils were well-to-do. I should think I was the poorest who went. Because her fees were very high’.^13^ These reflections are repeated at various points in the interview. This may in part be a result of Wooler’s age at the time of recording (she had just celebrated her ninetieth birthday), but it also stresses how deeply seared into old age these memories of elocution and class were for her. After her training, Wooler would provide lessons to others on the boundaries of the class system, including a variety of ‘lady mayoresses’ (likened by Wooler to ‘scared rabbits’) forced to do public speaking on their husbands’ behalf,^14^ and she also undertook the treatment of children who stammered.^15^

Of course, in advancing a set of arguments around performative class and dislocation, I do not want to overemphasize the ‘transgressive’ aspects of ‘passing’^16^ nor minimize the reality and consequences of the unequal distribution of wealth and capital by suggesting that class in twentieth-century Britain was wholly performative and based on external signifiers alone. Much of the earlier scholarship on class passing as transgression and embodied performance in the nineteenth century—for example in Seth Koven’s analysis of ‘slumming’ or Anne McClintock’s discussion of fetishized manual labour^17^—focused on concerns about the wealthy ‘passing’ down the social scale. More recent work on the twentieth century has inverted this gaze, examining concern around the ability of those at the bottom to imitate their supposed superiors. As Matt Houlbrook argues through the figure of the ‘gentleman crook’ Netley Lucas, the key to the latter’s confidence tricks lay in his ability to exploit the performative dimensions of (aristocratic) class identity. At this inter-war moment, Houlbrook argues that there was an anxiety around class as permeable and performative taking hold: a ‘broader crisis of confidence in everyday social relations’ and the ability to read social differences when learned mimicry confuses outward appearances.^18^ Acting and the training of the voice as a potential means of social advancement were a touch point of mid-century culture, but ones that could equally be satirized; for example, by Noel Coward’s injunction to ‘Mrs Worthington’ not to ‘put your daughter on the stage’.^19^ As we shall see in a later section, speech training critics of elocution were not averse to using elocution’s very performativity against it, especially when allied with misogynist discourses that cast aspersions on women’s abilities to speak properly. Performance, when exposed as performance, was key to the ‘legibility’ of class through accent.

The extortion of confidence tricksters might be one thing, but for the majority of people it remains to be seen whether the possibilities opened up by interventions to the speaking voice were liberatory. If anything, the dominant narrative—as in Wooler’s case—appears to be a sense of discomfort and alienation. Of course, consciousness of accent would not be the only cause of this, but it might well be the most immediately noticeable, particularly if it had been deliberately cultivated as part of an aspirational strategy. As Rebecca Conway has argued about Lancashire’s ‘Cotton Queens’, lessons in elocution and deportment for the winners of these accolades reveal ‘tensions’ both in the ideas of working-class authenticity that underlay their appeal and in the potential for these young women to return to factory life afterwards without a sense of dislocation or frustration.^20^ Contemporary voices, too, hinted at this. George Orwell, parodying Marx’s formulation, exhorted his middle-class readers to rally to the working-class cause with a direct interpolation of classed speech: ‘we have nothing to lose but our aitches.’^21^ Meanwhile, Richard Hoggart described—autobiographically and in the highly gendered form of the ‘scholarship boy’—the ways in which select members of the working class learned ‘to make use of a pair of different accents, perhaps even two different apparent characters’ in their enactment of social mobility.^22^

Accent and the voice were thus two key sites for experiencing—and even expressing pride in—class, as explored in Dave Russell’s and Patrick Joyce’s analyses of dialect publishing and northern civic culture from the nineteenth century onwards.^23^ Yet, Russell suggests, experiences of difference in interactions across geographical areas and class boundaries also reflect differences in representation, and the ability of accent to stand as a signifier for class beyond particular geographical confines. This is not simply a British story. As Josephine Hoegaerts’ work demonstrates, efforts to standardize speech are testament to pan-European, nineteenth-century efforts at state-building and reifying national characters, literary canons and linguistic borders.^24^ Hoegaerts’ approaches find resonances in Joy Damousi’s analysis of elocution and voice culture in modern Australia, which she locates in a transition from a project of British imperial and cultural hegemony to a new national standard oriented away from performative ‘Britishness’.^25^ Both scholars see, in attempts to define the ‘correct’ talking voice and improve its mechanical and resonant processes, the emergence of gendered norms and (through elocution) a space for women to partake in ‘public’ speaking.^26^ For Britain itself, Lynda Mugglestone’s comprehensive study, *Talking Proper* and Jurg Schwyter’s *Dictating to the Mob* (exploring the BBC’s Advisory Committee on Spoken English) examine in very attentive ways shifts in what constituted ‘correct’ English, tracing the emergence of ‘received pronunciation’ as a norm coded as ‘placeless’ to which all citizens could aspire.^27^ Indeed, it is inescapable that the reach of radio and cinema by the 1930s accounts for a higher degree of public consciousness of accent, a factor which may well drive the boom in elocution and training. Joe Moran, reflecting on the use of audio as an historical source in Britain, refers to how the emergence of ‘recording technologies … led to a renewed awareness of the voice as a trained instrument’.^28^

Yet, apart from the work cited above, historians of class in Britain have largely avoided accent and voice as markers of social status or a way of experiencing classed difference. Where they have engaged with this, its presence is often minimized, as in David Cannadine’s study *Class in Britain*, which devotes only a handful of pages to the subject.^29^ The suggestion appears to be that accent in the past was a more pronounced marker of class, in counterpoint to the mid-century, when—as a variety of historical and sociological analyses show—class identity became increasingly muddled and illegible^30^ (whether inscribed through the voice or otherwise). I would argue that this trend began earlier in the century, particularly in how speech trainers sought to make use of the welfare state and educational reform after 1945 to appeal for a new kind of national speech that would transcend class. These lofty ambitions did not materialize and, indeed, appear to have been critiqued from multiple angles. Not for nothing does Hoggart, perhaps the British intellectual whose work most epitomizes disaffection and dissatisfaction with the levelling potential of affluence, state in *The Uses of Literacy* that the real danger lay in ‘those major social developments of our time towards centralization and a kind of classlessness’: dangerous because they afforded no comforting identity and minimal consolation for alienation, rather than offering a truly liberating overhaul of social relations.^31^

This means that elocution has perhaps become something of a joke in Britain: what Moran, referring to perceptions of ‘stuffy’ ‘BBC’ accents has criticized as a ‘lazy shorthand for embodying social change’ which allows contemporary audiences to indulge in reverse snobbery;^32^ or what Damousi, describing similar attitudes to elocution in the Australian context, labels ‘a quaint notion’.^33^ This article follows Mugglestone, Damousi, Moran and Hoegaerts in suggesting that the history of speech matters. The speech movements under consideration here mattered in inter-war and immediate post-war Britain, and we should read the productions of their proponents as addressing questions of class within British society. Even if class was often submerged or approached only via proxy in their writings, this unease suggests a deeper concern with the possibilities—both political, social, and psychological—that class ‘passing’ offered.

## Elocution, Speech Training, and Speech Therapy

In order to make sense of how contemporary voice professionals related speech to class, it is worthwhile at the outset to disambiguate elocution, speech training, and speech therapy. To do this, however, also requires paying attention to their common ground and, indeed, the individual actors who passed between them. Elocution, as Marian Wilson Kimber argues for the US context, was largely a feminized and artistic endeavour inherited from mid-nineteenth-century performance and musical cultures. It typified the rise of an urbanized middle-class keen to integrate spoken word and musical performance into an enactment of cultural capital centred on a canon of suitable literary texts.^34^ Elocution teaching and performance were therefore by no means new in the early twentieth century. Yet, by the inter-war period, it was being taught in a much wider range of settings in Britain than at any point previously. It was also very rapidly becoming standardized and institutionalized through formal certification, graded examinations and common syllabi. All of these may have helped to contain its transgressive potential by making it openly a question of performance and art. For instance, speech therapist, writer of speech training manuals and overt enemy of elocution, Henry St John Rumsey, painted a derogatory picture of overly-ambitious aspiring actresses crammed into elocution schools hoping to emulate the careers of cinema stars but doomed to earn their living from ‘chorus work’ and the treatment of children’s lisps.^35^

Efforts to standardize elocution and drama training in Britain, at least in the later nineteenth century, had meant looking enviously to France and the diction training offered at the Paris Conservatoire under French elocutionists and actors such as Jean Coquelin and Henri Dupont-Vernon. Their approach stressed clarity and focused on reinforcing the musculature of the organs involved in speech. British proponents of these methods criticized their compatriots—British people in general, not just the working classes—for ‘mumbling’.^36^ By the early twentieth century, elocution courses were being offered at both the Royal Academy of Music (RAM) and its sister institution the Royal College of Music (RCM), as well as the newly-created drama schools: the Royal Academy of Dramatic Art (RADA) and the Central School of Speech Training and Dramatic Art (founded in 1905 and 1906 respectively). There was also a thriving local culture of elocution training, as Hilda Wooler’s experiences might attest. The RAM, the RCM and their joint board (the Associated Board of the Royal Schools of Music, or ABRSM) offered various elocution diplomas and graded examinations through a network of national examination centres, the latter launching in 1921. According to the ABRSM historian David Wright, the number of elocution candidates examined nationally in the inter-war period remained low, and was artificially suppressed by war conditions, but had risen to 4384 by the 1945–46 academic year.^37^

If music schools appear an odd location for institutionalizing elocution, this must be set within the twin contexts of the relatively late adoption of formalized drama training in Britain (as evidenced by the turn-of-the-century origins of RADA and the Central School) and the ways in which the environment and broader culture of the music school fitted with elocution’s mission to construct an exclusive literary canon and corpus of spoken English to match that of classical music.^38^ In the 1924–25 RAM ‘Licentiateship’ syllabus, candidates were expected to present four pieces including one prose (the candidate’s choice), one poem (nineteenth-century), extracts from a classical tragedy (Shakespeare or translated Euripides) and either eighteenth-century comedy or modern drama, including an option from George Bernard Shaw.^39^ (The latter author, an upwardly-mobile Dubliner who had presented his own take on the voice as performative instrument for social porousness in *Pygmalion*, was a champion of elocution and a frequently invited judge of elocution competitions and festivals, in addition to being chair of the BBC’s Advisory Committee on Spoken English.^40^) The graded examinations and competition culture, transplanted from the world of musicianship,^41^ also conferred cultural capital on students and teachers of speech alike. Interviewing Hilda Wooler, many decades later, the interviewer Benita Moore is shown and remarks upon Wooler’s impressive RAM certificates.^42^

The RCM merged its elocution and dramatic classes from the 1906 academic year^43^ and offered a specific examination in ‘Elocution and Declamation’ for its ‘Associateship’ diploma from 1925 onwards (due to a gap in the RCM Library’s holdings between 1906 and 1925, it is not possible to tell when precisely this began).^44^ However, the RCM’s elocution course was considerably more music-orientated than the RAM’s, which eventually sidelined any assessment of musical skill.^45^ The drama schools, meanwhile, saw elocution as a worthwhile money-generating exercise. RADA, from its foundation, stated that private classes in elocution were available for ‘those not wishing to make the stage their profession’.^46^ Elocution even enjoyed connections to avant-garde culture. Eve Acton-Bond’s ‘euchorics’, combining vocal and gestural exercises based on eurhythmics, was designed to instil children with a greater appreciation of the metre and emotive expression of poetry.^47^ Acton-Bond appears to have been the daughter of the (wonderfully named) Acton Acton-Bond, an early elocution tutor at the RAM,^48^ and she demonstrates how even the most apparently eccentric or esoteric forms of vocal culture were usually connected back to elocution’s institutional powerhouses.

Most revealing about the elocution story is how it not only parallels the growth of the discipline of ‘speech therapy’ but is implicated within it. Many therapists had been trained as elocutionists or teachers of speech first and foremost. Some either transposed themselves sideways into therapy, or used their voice training as a basis for further study. The music school curricula even reflected this: the RAM’s ‘Licentiateship’ examination for elocution (intended as an accreditation for teachers and open to external candidates as well as the Academy’s own students) expanded to include questions on the treatment of common ‘defects of speech’ and articulation errors in its syllabi from the 1920s onwards.^49^ Some elements of this continued until at least the 1950s. In the 1955–56 syllabus, the three-year diploma course—now rebranded as ‘Speech and Drama’ to reflect a shift towards speech training—included a lecture course on ‘Psychology’ and ‘elementary treatment’ for ‘Remedial Speech’.^50^ Early therapists recounted being persuaded to enter their profession through an enjoyment of the voice and involvement with music-making and amateur dramatics.^51^ Yet if the two were bound together initially, elocution rapidly became scientific speech therapy’s other—both in an intellectual effort to police the contours of the discipline and in the relationship between the two professions, accent and class.

Like elocutionists, the large majority of speech therapists were women, often from middle-class backgrounds, and they consequently had to negotiate everyday discrimination by male medical officers.^52^ Formally founded in 1945, the profession’s regulatory and examining body, the College of Speech Therapists (CST), was the product of delicate merger negotiations which had taken place during the Second World War between two rival professional groups: the British Society of Speech Therapists (BSST) and the Remedial Section of the Association of Teachers of Speech and Drama (ATSD).^53^ The former aspired to a scientific approach and largely brought together therapists trained in Scotland (under Anne McAllister in Glasgow) and those English therapists who had been through the speech course at the West End Hospital for Nervous Diseases. The latter were trained through Elsie Fogarty’s (1864–1945) course at the Central School of Speech Training and Dramatic Art and often had drama or elocutionary backgrounds. Despite their seemingly intense professional differences and rhetorical bluster, both sets of therapists had been exposed to clinical work and had received training in the physiology of the vocal organs and psychological theory. Even the usually staid minutes record intense acrimony and lack of respect at these meetings, particularly by the BSST members for their ATSD counterparts.^54^ Issues around the design of common training programmes and qualification routes into the profession were often the cause of these disagreements, but so too were less tangible concerns of status and proximity to medical authority. To combat these perceived training gaps, the newly-formed CST undertook several actions. Firstly, it began an immediate war against non-registered therapists (those who had not passed the College’s examinations) promoting themselves as being able to cure stammering and other ‘speech defects’. This put figures like Raw, whom we met at the start of this essay, firmly in their firing line. It also meant that some male and hospital-based therapists, like Rumsey, fell outside their scope, leading to mutual distrust and angry correspondence.^55^ Secondly, and perhaps more delicately, the CST began to criticize decisions by some local authorities to recruit non-registered practitioners (mostly elocutionists) to fill the large number of vacancies which the expansion of the school medical services after the Second World War required.^56^

As part of their training, speech therapists were obliged to pass an examination in ‘Normal Voice and Speech’ well into the 1970s. This was effectively an elocution course assessed through the standard of the candidate’s spoken-word performance. Manuals teaching this component of the course (such as Anne McAllister’s *A Year’s Course in Speech Training*)^57^ represent the third part of the voice profession triptych and the most difficult to pin down precisely: speech training. In some respects, this was largely a rebranded elocution and its pedagogical methods could include the recitation of poetry. What distinguished it, perhaps, was attention to the possibilities offered by the education system and the wider democratizing discourses of the welfare state. It was also typified by a desire to police boundaries, as the next section will demonstrate through attention to how proponents of all three approaches discussed class, local accent, dialect, geography and gender.

## Speech Training and the Welfare State: Dialect, Accent, Geography and Gender in Speech Training Manuals

For local speech therapists, the desire to downplay links to elocution and avoid class manifested in the ways in which they refused to engage in discussions around accent in their annual reports (collated as part of the School Medical Officer and Medical Officer of Health reports). Where local specificities were mentioned directly, the tone was often remarkably conciliatory and keen to avoid pathologizing specific accents. This choice was undoubtedly strategic, but it is highly revealing that such disavowals of class in speech therapy only appear in reports from predominantly industrial, working-class areas and ally locality with the psychosocial to make the case for or against medical intervention. In one exceptionally ponderous reflection in their 1955 annual report, the Leicestershire speech therapists admitted to being ‘frequently asked what exactly is the scope of our work’. Their response noted, as though this were a common misconception to correct, that it was treatment for a child ‘whose speech deviates from that which is normal *to his environment*’ (their emphasis) and that ‘these latter words are important as we would not attempt correction of a local dialect, unless we discovered that consciousness of dialectal speech was a contributory cause for a speech disorder’.^58^ Dialect was thus deliberately elided as something problematic or difficult, only shading into pathology when it caused distress.

Localities and their specificities were often used as a substitute for writing about social groups, fixing the problems firmly in geography rather than class. In 1952, Caernarfonshire’s therapist reported finding a:

quite remarkable increase in lisps and defective R sounds, as I progressed towards the Llandudno end of the county. This was most marked and was especially noticeable among the Grammar School children and those coming from the better class homes. Many parents considered it an added attraction, and I feel this attitude was probably largely responsible for the defects.^59^

Class pathologization was inverted here, with parents criticized for encouraging accents with falsely aspirational associations. (The ‘R sound’ referenced here is probably not so much the Welsh ‘rolled’ R but rather an imitation of English Received Pronunciation producing a soft, almost W-sounding, R.) Other cases explicitly identified by therapists at this time were the product of geographical—rather than social—mobility. The speech therapist in another Welsh authority, Port Talbot, noted that her large number of ‘dyslalia’^60^ cases included three children (one Scottish and two English) ‘who had only recently moved’ and who ‘all had marked dialectal variants in their speech’. This therapist took comfort from the psychological observation that ‘[a]ll normal children are anxious to conform with the accepted speech of the area in which they live’, though her wording suggested that only a professional might be able to ascertain where the line between one ended and the other began. The children’s new environment was felt to act as a mirror, allowing them to become ‘increasingly aware of their own speech’ differences—whether a result of dialect or pathologized articulatory faults—and thus improve.^61^ Notably absent here was the idea that the speech therapist either could or should change the child’s accent, even though this was acknowledged (as in the Port Talbot case) to be malleable and environmentally contingent.

If the accents of England, Wales and Scotland could enjoy some level of coterminous respect, the exception was Ireland. Although Belfast provided speech therapy through its child guidance clinic from the late 1930s, County Armagh was the first authority in Northern Ireland—and, its School Medical Officer claimed with pride, the first on the island of Ireland^62^—to undertake a full survey of the school population and tabulate the incidence of speech disorders found. Led by the English speech therapist, Edna Butfield, a key figure in the development of the CST, the final report compared incidence of ‘speech defects’ in County Armagh (surveyed in 1949–50) to the London borough of East Ham (surveyed in 1946–47). Butfield found a much higher incidence—in fact, a doubling—in cases between the Northern Irish county and the metropolitan area. Whereas 0.6 per cent of the total school population were found to stammer in East Ham, while 1.3 per cent of children exhibited ‘articulation defects’ (another term for dyslalia), the respective rates for Armagh were 1.4 and 2 per cent of the school population.^63^ Yet, even here, direct interpolation of class was elided. Butfield blamed speech pathology largely on the children’s rural existence and the broader pathology of locality and family life. Not only did they live ‘in remote areas where vocabulary is extremely limited, and the speech pattern of the family primitive’, she claimed, but the ‘religio-political tensions’ of the province and parental pressure for ‘high educational’ achievements suggested a more psychosocial causation.^64^

Butfield’s survey demonstrates the extent to which, even in an area where the accent was so geographically distant that it could easily become an ‘othered’ voice, there was a conscious shift to thinking in a language of familial transmission of speech disorders. This transferred the burden for causing vocal abnormalities onto the child’s parents and allowed therapists to problematize locality, and the psychological relationships within families, without mentioning class. According to the Dagenham therapist, Eileen Mills (for whom ‘articulation’ disorders were ‘very much my field’), part of the joy came from the audibility of progress, provided parents ensured regular attendance. But, Mills claimed post-retirement in 1995, ‘I saw people into the second generation, even … the third generation of speech defects, because speech defects can be strongly familial and get passed on’.^65^ In imagining their differences from elocutionists, early speech therapists were thus forced to navigate ways in which they could blame locality and family without introducing ‘class’ as an analytical frame. There may have been a sense of speech therapists experiencing their own class abjection here. In her oral history research with successive generations of retired therapists, Jois Stansfield has noted that many of her interviewees often spoke with RP accents but claimed working-class or socially mobile backgrounds. As the existence of the ‘Normal Voice and Speech’ examination shows, therapists’ own voices were thus often the product of deliberate intervention and training. Some of Stansfield’s interviewees reported this as an undesired intrusion, but one which seems to have offered some opportunity for professional advancement.^66^ As we shall see below, therapists who also sought to engage with speech training had to confront similar issues.

Those engaged in speech training shared the speech therapists’ desire to avoid judgement and distinguish dialect from accent. But they also appealed directly to the welfare state and the new education system, imagining it as a tool to spread ‘good’ speech to all children. This language of ‘good’ speech steered a very clear course from stating what ‘bad’ speech might look like. The 1921 Newbolt Report into the education of English had placed a premium on literature and poetry to be experienced through recitation (as a tool to ‘improve’ pupils’ spoken English).^67^ Indeed, some of the texts considered in this section were intended as textbooks for this. In the aftermath of Newbolt, some elocutionists, such as Vera Beringer of the RAM, did lobby for a formal spoken English examination in the School Certificate (the end of secondary school diploma). In 1939, she contacted various government officials, lamenting the low ‘standard in this country’ for speech and claiming health benefits from the ‘correct breathing’ taught by speech training.^68^ Unfortunately for Beringer, civil servants at the Board of Education were swift to reject the idea of an oral examination, citing administrative issues.^69^

Some writers believed that the post-war welfare state offered the optimistic promise of a space for a new kind of speech training, democratized for all citizens, which did not carry the baggage of inter-war elocution. In the process of articulating their visions, however, they had to confront the same issues around class as speech therapists, equally employing locality and geography as substitutes. Some were even able to mobilize the psychosocial language of the speech therapists considered above, to place the need for national consistency in speech alongside the preservation of local variation, usually claiming that this would avoid a sense of cultural dislocation for the child. For instance, Rose Bruford—briefly an elocution teacher at the RAM and later founder of her own drama school^70^—argued that ‘good speech’ was not necessarily opposed to dialect. Yet, as she wrote in a 1948 book advising schoolteachers on speech training for children, developing clear and well-articulated voice production was beneficial for both ‘aesthetic’ and ‘psychological’ reasons in social relations: it allowed the individual to form a ‘good relationship’ with others ‘without any feeling of inferiority’.^71^ The important aspect to speech training in this conception was a democratic focus on mutual intelligibility, or ‘clarity’, which Bruford suggested was in tension with *dialect* (as a localized idiom with its own vocabulary, syntax and grammar) but not necessarily *accent* (which, as she and other contemporaries reminded their readers, was chiefly located in the vowels, and only to a lesser extent in the consonants, of English speech). Even so, teachers were encouraged to not ‘eradicate’ dialect, with Bruford warning of the harmful psychological consequences of ‘increased self-consciousness’ for children and adults alike.^72^ This is similar to what McAllister considered the benefit of standard speech in enabling a person ‘to speak without embarrassment or self-consciousness in any company, or in any class of society in which he may find himself’.^73^

These appeals to equality were not always shared. Interestingly, it was often older generations of men who disagreed with female professionals. Rumsey, coding this in the aspirational language of secondary schooling, warned of the dangers for the ‘average boy passing out from the secondary schools’ searching for clerical work and finding that the ‘better speaker is getting the job … over the more learned boy with a strong provincial accent’.^74^ Bruford’s former RAM colleague, Geoffrey Crump (who taught from 1949 until the Academy’s ‘Speech and Drama’ course was discontinued in the 1960s), would have been inclined to agree with this in his guide to teaching verse-speaking and poetry recitation published five years after Bruford’s. While regional variations were ‘serviceable’, at least ‘for the purposes of ordinary speech’, Crump claimed that ‘a so-called standard pronunciation’, ‘based on southern English’, was more ‘desirable’ as an adopted performative mode to be taught in the classroom.^75^ The assumption in all these cases, however, was that a social democratic nation required standards which functioned as a *lingua franca* and could consequently facilitate access to the nation’s literary and poetic canon (even as it continued to uphold the elitism inherent in the notion of a canon). Such shared modes of speech would increase an egalitarian sense of shared identity, but—by preserving something of the regional and localized features of their place of birth—would not force children into transgressive acts which made background difficult to parse from speech alone or produced unsettling feelings of dislocation from their immediate family and friends.

If elocution in the inter-war period was marked by the possibility of dangerous transitions and ‘passing’ across class boundaries, its post-war iterations sought to contain this radical potential. Bruford and Crump did this by subtly, and perhaps rather disingenuously, disavowing the ‘superiority’ of one accent over another. Others instead laid stress on the ‘vitality’ of voice and claimed a naturalized manner of speaking against an over-performed tone that might veer into parody. Harold Ripper, author of the 1938 volume *Vital Speech—*in the sense of speech ‘full of life’ rather than ‘necessary’—claimed that elocution had actively harmed the voice. In a remarkable reversal of the classed gaze carried out through a gendered reading, Ripper targeted female BBC announcers, whose vowel sounds were ‘“decorated” out of shape’ and whose ‘tone’ was ‘to the sensitive ear … far more ugly than the robust tones of the peasant, or even of some town-dwellers’.^76^ Gender, combined with over-elocution, had produced speech that others, including Rumsey, decried as ‘refaned’ or ‘raffeened’ (that is, a corrupt and parodically over-exaggerated pronunciation of ‘refined’).^77^ Ripper, like Rumsey, was one of a minority of men in the speech therapy profession, and his approach suggested a post-war emphasis on ‘authenticity’ in accent (speech ‘full of life’) rather than one that was merely a superficial or performative mask.

Unlike many of the female therapists, Henry St John Rumsey (1884–1972) classified himself as a ‘self-cured’ stammerer who had subsequently moved into the therapy of others. In this, he was following a common route at the end of the nineteenth century, albeit navigating a transition to medical respectability better than some of his predecessors.^78^ Attending King’s College, Cambridge, as a choral scholar, he described his moment of vocal epiphany as the realization that he did not stammer when he sang, and therefore that the key to fluent voice production lay in breathing and articulation. Having ‘cured’ himself of stammering, he set about treating fellow undergraduates, and later pupils during a short career as a teacher in a fee-paying school.^79^ In 1923, he was appointed speech therapist to Guy’s Hospital in London, working under its Ear, Nose and Throat department, and later became one of the first therapists to be formally appointed as a ‘Lecturer in Speech Therapy’ through the hospital’s teaching division.^80^ Self-assured of his own genius, in 1945 Rumsey refused to recognize the authority of the newly formed CST and to pay a five guinea registration fee, despite the organization opening a special route that allowed (mostly male) therapists like him whose training predated even the formation of BSST and ATSD to circumvent the qualifying examination.^81^ His writing subsequently took on a markedly more misogynistic and hostile attitude towards the speech therapy profession, although he had argued throughout the inter-war period that only men who were ‘self-cured’ of stammering could engage with male stammerers in any meaningful therapeutic relationship.^82^

Rumsey’s prolifically published speech training manuals often took issue with elocution, expressly gendering it as female in contrast to male speech training. He simultaneously criticized its claims to authority over voice production and targeted its more performative dimensions. In 1936’s *Your Speaking Voice and Its Possibilities*, Rumsey accused elocution teachers of having only ‘taught the veneer of’ good voice production. He noted that it was ‘far easier and far quicker to teach mannerisms than to teach voice production, but no number of clever tricks can conceal faulty tone production’.^83^ The promise of speech training was thus that it could detect the falseness of elocution and undermine the ability to ‘pass’. In the following year’s *Speech Training for Children*, Rumsey identified the tell-tale sign of poor elocution-based training as ‘the strangling of vocal tone by the exaggeration of the word formation’, particularly through ‘the exaggeration of consonants’—a trait that he blamed on parental encouragement (especially from mothers).^84^ Like Ripper, he took aim at ‘the insistent lisp heard when trained women speakers talk on the air’, another supposed pretension from their elocution training.^85^ It was precisely because elocutionists did not teach correct consonant and vowel production ‘of what we may call Southern Educated English’ that he warned stammerers against seeking any advice or treatment from them. His criticism went back to the professionalization of elocution itself and its institutionalization:

With the enormous expansion of elocution as a business there has come into being an examination system whereby pupils can gradually progress from diploma (in various grades) to medals (in three grades) and finally to a teacher’s diploma. This diploma generally contains a statement to the effect that the holder is qualified to undertake the correction of speech defects and disabilities, but as far as can be ascertained, among the various schools of elocution which organize public examinations and grant diplomas to teachers, there is only one in which instruction in speech therapy is given by a specialist in that work and who holds a public appointment.^86^

Rumsey’s critique of elocution lay in its very artifice and performativity; that it provided a ‘veneer’ rather than substance. However, the real risk lay in its ability to expose the speaker as someone engaging in a performance. This was particularly dangerous with what Rumsey termed ‘[c]orrected dialect’ which was ‘useless unless it is spontaneous, because in moments when it is most important, it may fail’.^87^ Many of these issues came from false imitation for the sake of social mobility or perceived cachet, much like the boy who has won a scholarship and ‘imitates the speech of those in the grammar school’, Rumsey claimed.^88^ He was ferocious in his demarcation of ‘the difference between teaching recitations to children and the art of clear speaking to men and women’, which, for him, marked ‘the difference between elocution and speech training’.^89^ For Rumsey, the gendered subject receiving therapy and benefitting from speech training was nearly also always male or in a profession that could be coded as male in the reader’s mind. Conversely, it was nearly always women whom Rumsey accused of poor voice-production techniques, even where—he noted derisively—they had ‘an attaché case filled with elocution diplomas’.^90^ It was the female pronoun he employed when he warned stammerers not to seek help from an imagined elocutionist.^91^ It was also women who, he argued caustically, had turned elocution into an exploitative ‘business—I will not call it an art—of learning to exaggerate consonants during recitations’.^92^

Speech training, in Rumsey’s analysis, was therefore not solely a question of rebranding elocution for the welfare state. It was equally implicated in a (barely concealed) misogynist project of gendering expertise around the voice and wresting control of female-dominated fields such as elocution and speech therapy. Gender and family relations served the role that geography played in Bruford’s work. One thing that Rumsey had in common with Bruford and Crump, however, was that they were all writing from an *English* (and largely London-based and metropolitan) common understanding of what ‘good’ and ‘standard’ English should sound like—something typified in Rumsey’s preferred phrase of ‘Southern Educated English’, which concealed class behind both educational elitism and geographical designation. The appeal beyond class towards geography proved more strained (as in Butfield’s example) for those located on the geographical margins of ‘Britishness’ and who were more conscious of the othering potential of interventions to change accent. One example of this latter group was Anne McAllister (1892–1983).

A Scottish phonetics scholar who obtained her PhD from the University of Glasgow, McAllister became interested in articulation by children recovering from cleft palate operations. In the 1920s and 1930s, she provided speech therapy services to Glasgow’s School Medical Service before establishing her own school for training therapists in the city. Between 1936 and the outbreak of the Second World War, McAllister also coordinated and presented a series of experimental broadcasts in speech training for schoolchildren—at junior and senior level—through the BBC’s regional service for Scotland. The advisory board for this project also included the English phonetician (and active member of the BBC Advisory Committee on Spoken English) Arthur Lloyd James^93^ and various Scottish dignitaries, religious leaders and academics. Although, regrettably, no recording survives, McAllister used the published booklet that accompanied the series of talks and interactive activities to defend the linguistic choices of the programme against accusations of Anglicization, possibly reacting against memories of older efforts to instil English speech as a norm in Scotland identified by Mugglestone.^94^ As was common in speech training methods, physiology was to go together with literary appreciation: exercises involved practising recitations and vowel and consonant sounds, as well as learning about the structure of the larynx and techniques for mouth and tongue movement.

The introduction to the senior workbook, directed to teachers rather than pupils, justified the training with reference to what was outside its scope as much as to what was within it. McAllister demarcated a difference between the total elimination of (Scottish) regional dialects or accents and the correction of ‘faults of indistinctness and unintelligibility’ which might pose a barrier to effective communication. ‘There is’, McAllister claimed, ‘no single form of speech which in Scotland is universally regarded as “correct”’, and provided ‘[d]ialectical characteristics’ were ‘not too inacceptable for social or vocational reasons, the Committee see no reason why a Speech Training course should seek to eliminate them or … impose any arbitrary standard’.^95^ Teachers were told in the Junior level workbook that ‘consonant formation will be dealt with very simply without any specification of local peculiarities’ with remedial effort mainly ‘directed towards eliminating clumsiness in consonant coordination’.^96^ That McAllister even hoped to enlist teachers into corresponding with the committee on local vowel variations, culminating in an unofficial survey of the state of Scottish speech, does not suggest a desire to eliminate these local variations as incorrect.^97^ The children themselves, in this case the Juniors (aged seven to nine), were exhorted to see the benefits of good speech as both utilitarian and aesthetic; they were told in the pupil-facing parts of the workbook to ‘remember that it is *useful* to be able to speak clearly’ and reminded that this kind of speech ‘pleases not only yourself but everyone who listens to you’.^98^ Such appeals placed a duty of clarity within a broader framework of good citizenship and anticipated post-war developments.

Although there is no evidence that this course had any retransmission elsewhere in Britain, McAllister did adapt the training manuals and pupils’ workbooks before and after the Second World War as *Steps in Speech Training* and *A Year’s Course in Speech Training* (the latter intended to help speech therapy students prepare for their Normal Voice and Speech examination). The former was organized in a series of graded levels, first published between 1938 and 1940, which appear to have been reissued regularly until the 1950s. Because these were intended for use on either side of the Border, many of the efforts taken to legitimize the course through reference to specificities of Scottish speech were deleted. However, they were not removed entirely and McAllister included notes for teachers on differences between ‘English’ and ‘Scottish’ pronunciation which avoided being overly judgemental about which norm was correct.^99^ Occasionally, a specific exercise would require that some form of localization had to be recognized, usually as incorrect, and subject to attempted modification. This was usually prefaced by noting that ‘as many English as Scottish localities’ employed the fault and that the aim of the exercise was to create a shared norm rather than pathologize local particularities.^100^ McAllister had to go to great lengths to appear fair and even-handed, suggesting an awareness of potential resistance.

As in speech training more generally, ‘dialect’ and ‘accent’ were separated from pathology or defect. McAllister told teachers quite bluntly that ‘the speaker of a local dialect can produce his voice as pleasantly, … as the speaker of so-called Standard English’ and that ‘the use of a local dialect is quite compatible with *good speech*’.^101^ In this framework, as we have seen already, ‘articulation’ and ‘clarity’ took the place of accent in delineating ‘good’ speech as that which could ‘be easily followed by the hearer’.^102^ Speech was contextual in any judgement of its quality. It had to be intelligible to the widest number, and faults could be found, McAllister claimed—with an eye (or ear) on a Scottish audience—even among the speakers of the most pure, ‘much-lauded Southern English’ who must ‘yield place to the speaker of the local dialect who frames his native vowels in an accurate and clear-cut articulation’.^103^ McAllister reconfigured dialect on rural–urban continuum: the former having the purest forms of dialect (represented, for her, by English-speakers in the Scottish Highlands), the latter being inherently more disordered and pathological (if never geographically identified precisely). Referring to speech training for primary school-aged children, she wrote that the only ‘reasonable attitude to local dialect speech’ was that it ‘must not be decried’, laying stress on a discourse of ‘purity’ as a marker of authenticity. But, she followed disapprovingly, ‘in our day very little pure dialect is to be found even in the country districts’.^104^ The majority of children spoke ‘not a dialect but a degenerate and slovenly form of English to stamp out which every effort should be made’. The onus for this latter was to be placed very definitely on the teacher, since little good could emerge from the home and parents who had likely transmitted such pathologized dialects to their children in the first place.^105^

This meant that while pupils were not to have their dialects subject to relentless attack and criticism, a teacher ‘should attempt to discard the more peculiar marks of local dialect and to use a pronunciation as near to the accepted standard as possible’.^106^ This sense grew during the post-war period, with McAllister arguing by the ninth edition of her *Year’s Course* that ‘intelligibility’, the real ‘criterion of judgement’, ‘can generally be ensured when the speaker purges his pronunciation of all variations which are distinctively local, and which are appreciated only in the place of their origin’.^107^ In tandem with this, and as with the Caernarfonshire therapist quoted above, McAllister tended to shift the blame away from class as a construct or structure, individualizing the pathological voice through reference to the family and home environment. ‘The homes from which some pupils have come may have made them unresponsively “speechless”’, she warned primary school teachers. Such children were ‘unaccustomed to the use of speech as a vehicle of self-expression or of instructive communication’. At the same time, she problematized the opposite, middle-class, issue of over-indulged children being given to ‘precocious talkativeness’.^108^ Speech training, as opposed to elocution, offered a way to rectify this, and ensured that future generations did not develop such problems. Speech training offered a third way between the excesses of elocution and the scientific focus on outrightly ‘pathological’ communication dealt with by speech therapy. It sought to employ the latter’s more scientific methods to the inculcation of ‘good’ speech, albeit without the taint of class passing and performativity associated with elocution. Indeed, while the CST was hostile to non-qualified elocutionists in local authority employment, it does appear to have turned a blind eye to the employment of speech trainers to complement therapy and to deal—in the words of the Stockton-on-Tees School Medical Officer’s report from 1950—with ‘non-medical cases of defective articulation’ (such as imitative dyslalia or lisping) whilst reserving to the therapist ‘those cases which can clearly be classed as medical’ (like stammering or cleft palate).^109^

McAllister’s use of domesticity as a way of circumventing class was similar, but also different, to Rumsey’s deployment of gender to code the pathology of speech. After all, Rumsey, as a male writer who fell outside the highly feminized profession to which he aspired, was using this to vent his own misogyny and present male voices as the norm from which female voices pathologically deviated. In concluding this section, though, it is worthwhile cycling back to Rumsey as a man who typified the fundamental paradox in the market for speech training manuals. While McAllister’s textbooks could at least benefit from sales to speech therapy students anxious to pass ‘Normal Voice and Speech’, Rumsey’s writings trace a line from optimism to pessimism in the possibilities of speech training. Initially hopeful that systematic training might be instituted through the schools, Rumsey was sufficiently disabused of this notion by 1950 to declare bluntly, in the second edition of his *Speech Training for Children*, that austerity meant that ‘much of the required speech correction will have to be done at home if at all’.^110^ This sense of state retrenchment may well have motivated Rumsey to target parents and adults who stammered. One of the main revisions between the two editions of Rumsey’s *Speech Training for Children* appears to be a more explicit focus on the parent as a user of the manual. ‘What the new under-planned system [of secondary education] is not ready to do’, argued Rumsey in the second edition, ‘parents must do at home; it is up to them to counteract the influence of a low standard of speech on the one hand, and to back up and help the child to acquire a better standard on the other’.^111^ By 1957, this had taken on a much more pessimistic tone altogether, concerned that the national standard of speech had suffered a ‘deterioration’ and a return to the excesses of elocution as ‘artistic rather than effective speech’.^112^ This may explain why, unlike McAllister, Rumsey did not view ‘dialect’ on a continuum of good or bad speech, although he did criticize affected speech imitating American film stars which he claimed had perverted localized varieties of English (mirroring Damousi’s analysis of similar anxieties in Australia).^113^ Instead, Rumsey believed dialects to be useful for their local and antiquarian value, but saw only ‘Standard’ or ‘Southern Educated English’ as having sufficient geographical range to ensure common intelligibility.^114^ ‘Dialects have an interest and charm of their own’, he wrote in a 1957 treatise on stammering; ‘but so have village cottages with tiny windows heavily screened with ivy, but the people who are most enthusiastic about dialect do not use it, and the people who photograph village cottages do not live in them’.^115^ This juxtaposition of images—a rural England contrasted to rational modernity—reflected a biological discourse which sited the voice as something requiring both ‘efficient’ use and skilfully training by those adopting physiological, rather than artistic, approaches.

## The Decline of Elocution

If speech therapy succeeded in establishing itself as an auxiliary profession in the post-war NHS,^116^ the moments of elocution and speech training waned. The period after the 1950s was marked by their gradual decline, particularly of commercial and institutionalized elocution so thoroughly critiqued by Rumsey. Cicely Berry (1926–2018), who had trained at the Central School under Fogerty and later had a successful teaching career there, shifted speech training towards ‘voice work’ in drama pedagogy. For Berry, a lifelong socialist,^117^ the class dimensions of vocal differences were to be resolved through improving delivery rather than accent. Accent training was to be used solely for actors to enable them to play a diverse range of roles. As Berry argued in a 1975 manual (whose title, crucially, did not mention either ‘elocution’ or ‘training’ of any kind), ‘a lot of voice work in the past has been done from a negative point of view’, characterized by ‘emphasis … on “correcting” the voice and making it “better”, with all the social and personal implications that the word “better” contains’.^118^ Voice was so intimately bound up with the individual’s identity that ‘[n]o voice is wrong if it is communicating adequately […] You cannot think of the voice apart from the person—it *is* the person speaking’.^119^ As Damousi notes, referencing Berry’s influence at the end of her own volume on voice culture in Australia, the overriding ‘move in the latter part of the twentieth century was towards an improvement of individual expression’ rather than the imposition of a shared collective style.^120^ To a certain extent, Berry was reading the runes in broader cultural shifts. The 1960s and 1970s made provincial working-class cultures (and accents) more mainstream and placed them at the centre of national culture as opposed to its periphery, whether in popular music or social realist cinema.^121^ Regional accents could even become aspirational in their own right, as Mugglestone notes through the rise of ‘Estuary English’ as a new southern standard after the 1980s.^122^

Meanwhile, the ABRSM rebranded its graded ‘Elocution’ examinations as ‘Speech and Drama’ in 1950, reflecting similar changes in the titles of the music schools’ own elocution diplomas. While this may have been cosmetic and left the content of the diplomas largely unaffected, the music schools progressively began to diverge from the elocution and speech training market. The RCM’s dramatic class was eventually subsumed into a general ‘Opera and Drama Training’ division by 1961 (once again, a gap in the prospectus series between 1959 and 1961 makes it difficult to discern when precisely this shift occurred), placing more emphasis on the musical application of dramatic skill than recitation and prose performance.^123^ The RAM likewise discontinued its course in the early 1960s as it decided to focus purely on music training.^124^ Finally, in 1986, the ABRSM graded examinations became ‘Speech & Drama, Acting & Communication Skills’, including a portfolio of different awards such as ‘English as a Second Language’, ‘Solo Acting Syllabus’, and later a ‘Spoken English’ examination. The first two grades of the latter were ‘designed for candidates of any age whose mother-tongue is not English’. Part of the assessment obliged the candidate to give a short ‘talk’ on a topic and then engage in extempore discussion with the examiner. This new format was finally discontinued, according to Wright, in 1990.^125^

As the diverse range of awards that eventually replaced ‘elocution’ and ‘Speech and Drama’ testify, the examined voice was increasingly being subjected to efforts to promote fluency under the motive of ‘key skills’ rather than accent or dialect ‘correction’. Speech, as Berry promised, was now contextualized, with a stress on ‘communication’ for business or education, rather than performing to fixed standards within the parameters of an exclusive literary canon. The focus on non-native speakers of English from this time also strongly suggests that the examined voice had become less a tool of class policing and instead a means to demarcate ‘foreignness’, and ensure its assimilation, in an increasingly post-colonial and multicultural Britain. However, it is also sobering to note that the spaces opened for working-class culture in the 1960s and 1970s did not remain. Recent concerns suggest that opportunities for working-class actors, and even chances to perform working-class roles, are disappearing under a recentring of middle-class representation (though this is somewhat nuanced by socio-economic analyses of the acting profession which suggest that there has never been an era of ‘meritocracy’ in acting).^126^

## Conclusion

By tracing the histories of speech training, speech therapy, and elocution through their interconnections, this article has made a case for considering them as continuous movements which can reveal much about shifts in the relationship between class and the voice in Britain during the middle years of the twentieth century. All proponents of speech training appear to have acknowledged the post-war settlement as offering the promise to democratize ‘good’ speech through schools. Most remarkable in these texts is the degree of welfare state consciousness; the idea that the child might, as a consequence of their public education, have an entitlement to access ‘good’ speech as much as they could expect to learn to read and write. Yet the kind of ‘good’ speech proposed was to be defined not by class or dialect but by ‘clarity’, with speech training’s proponents seeking the progressive convergence of regional variation into common forms. In doing so, speech training counterposed itself to elocution while drawing from speech therapy’s scientific understanding of vocal production. It was to offer no transgressive potential for upsetting class hierarchies.

There were always tensions within this project. Firstly, the key problem for speech training’s proponents lay in adjudicating where the demarcation between dialect and standard English should come. This is articulated best by McAllister, as a Scottish writer, in her refusal to laud ‘Southern English’ as the norm to follow, whilst equally realizing that the need for a standard required a demarcation of difference. For other writers, such as Rumsey, gender rather than locality became a more serious marker of vocal difference in the post-war period. Rumsey’s attacks demonstrate a marked gendering of elocution as female, theatrical and parodied, with his own muscular approach to speech training offering a masculine alternative. Secondly, the performative nature of class through the voice became something to avoid as a subject of direct acknowledgement in published texts. For speech therapists, this was clearly marked during their period of professionalization in the 1940s and 1950s. Speech training manuals, too, often made a performative disavowal of class and of the potential for their methods to facilitate passing between different classed identities. Of course, it is difficult to tell how far readers were conscious of this disavowal or actively tried to read around it. As John Gallagher notes with regard to the very different subject of early-modern foreign language and conversation manuals—themselves part of an ‘extracurricular economy’ like elocution—it is difficult to reconstruct the ‘use’ of educational texts as material and practical artefacts.^127^ How many readers simply skimmed authors’ injunctions (albeit often contradictory) about the supposed equality of different dialects to reach the chapters of consonant and vowel tables, and their associated exercises, which often favoured ‘Southern English’? Published material likewise elides the private opinions of therapists. Stansfield, through oral histories with retired speech therapists, has identified several mid-century cases of individuals changing their accent in order to conform to a perceived professional ‘norm’ of RP.^128^ Another therapist reflected on a case she had treated where the child patient was increasingly taking on the therapist’s accent, something which gradually isolated the child from her geographical community.^129^ These suggest that there was more open acknowledgement of accent and its politics among speech therapists outside the public-facing world of publications and reports and that oral history will be a very useful resource for placing these private attitudes within a broader history of voice practices at the end of the twentieth century.

Class, of course, never was (nor is) wholly reducible to external signifiers, and *perceptions* of class boundaries being fluid at a given time do not imply a correlated social reality of classless utopia or free passage for expression. It is telling, in both Houlbrook’s example of Netley Lucas and the elocution and speech training cases I explore here, that concerns about this fluidity usually emerge from the top of the social order (as with Rumsey). Experience of permeable social hierarchies through any combination of speech therapy, speech training, or elocution may well have been quite different if viewed from the perspective of those ‘below’; that is, those who actively sought out guidance on altering their spoken voice and were either disappointed in the results or found only discomfort and dislocation awaiting them on the other side. Instead, I would suggest that paramedical figures like Raw (with whom this article began) and elocutionists more generally were perhaps better placed to navigate and work within these broader cultural ambivalences around class for commercial purposes, appreciating both the potential and the danger in what they were undertaking. In contrast, speech therapists and speech trainers were more disturbed by it and wanted to deny its potential. Like all abject spaces in Butler’s sense, elocution was consequently subject to social censure and efforts to police its entry and usage, of which its rebranding and absorption under speech training was perhaps the most evident. What is often striking in these cases are the ways in which attempts to control elocution, far from emanating among left-wing commentators decrying bourgeois voice culture, were often from conservative or traditionalist figures, whose critiques (like those of Rumsey) frequently centred on its very artificiality and performative—almost theatrical and parodic—dimensions.

Raw ended his mid-1930s advertising copy with the promise that ‘[f]reedom is within your reach’—referring to liberation from both stammering and the classed, geographical markers of the voice itself.^130^ Biographical information about Raw—indeed, how ‘free’ his own voice may have been—is particularly scarce, and it has only proved possible to trace him through his self-promotional ephemera. The exception to this appears in 1931: ‘Stewart Drayton Raw’, amateur conjurer, sued a fellow magician for stealing one of his card tricks.^131^ The judge drew particular enjoyment from the trial, with demonstrations of the offending illusion descending the courtroom into farce. The comedy of this vignette aside, it serves as a reminder—if this is indeed the same man with a side hustle—that both the use of the voice and claims to professional authority over its correction were always, in part, performances; ones which, in mid-century Britain, often attracted showmen who promised, but may not always have delivered, transformative illusions of ‘freedom’ in speech.

## Footnotes

1
*Leeds Mercury*, 15 February 1936, 2; *Portsmouth Evening News*, 12 April 1937, 6.

2 Wellcome Library (London), P11749, S. Drayton Raw, *Speech Correction for Stammerers* (London, n.d. [*c*.1930s]), 3–4.

3 Conor Heffernan, ‘Desirable Bodies and Eugen Sandow’s Curative Institute in Edwardian England’, *Social History of Medicine*, 35 (2022), 195–216.

4 Roy Porter, ‘The Patient’s View: Doing Medical History from Below’, *Theory and Society*, 14 (1985), 188; See also: Roy Porter (ed.), *Patients and Practitioners* (Cambridge, 1985).

5 Josephine Hoegaerts, ‘“Is it a habit or is it a disease?” The Changing Social Meaning of Stammering in Nineteenth-Century Western Europe’, *Terrains & Travaux*, 23 (2013), 17–37; Josephine Hoegaerts, ‘Stammering, Stuttering and Stumbling: A transnational History of the Pathologization of Dysfluency in Nineteenth-Century Europe’, *Journal of Interdisciplinary Voice Studies*, 5 (2020), 129–46. See also: Denyse Rockey, *Speech Disorder in Nineteenth Century Britain: the History of Stuttering* (London, 1980).

6 Raw, *Speech Correction*, 6.

7 Raw, *Speech Correction*, 5.

8 Speech therapy’s concern with organic and physiological conditions, such as cleft palate, was the one exception here, of course.

9 University of Strathclyde Library Special Collections (Strathclyde), JCE/14/19/1/6, *Speech Training: Broadcasts to Scottish Schools: Summer Term 1935*, 4; Geoffrey Crump, *Speaking Poetry* (London, 1953).

10 ‘Voice’ and ‘speech’ are not strictly synonymous, even less so with ‘accent’. However, the ways in which these terms bleed across within the source material and popular discourse reveals much about how ways of speaking are bound up with identity and class in Britain.

11 Judith Butler, *Bodies That Matter: on the Discursive Limits of Sex* (London, 2011), xiii–xx.

12 Lancashire Archives (Preston), NWSA/MOORE, Hilda Wooler interviewed by Benita Moore, undated [November 1993], 14:00.

13 Lancashire Archives (Preston), NWSA/MOORE, Hilda Wooler interviewed by Benita Moore, undated [November 1993], 19:15.

14 Given her geographical area, it is very likely that at least some of these women were the working-class wives of local Labour Party councillors.

15 Lancashire Archives, Wooler interviewed by Moore, 03:00 and 17:00.

16 Peter Stallybrass and Allon White, *The Politics of Poetics of Transgression* (Ithaca, 1986).

17 Anne McClintock, *Imperial Leather: Race, Gender, and Sexuality in the Colonial Contest* (New York, 1995), chapter 2, ‘Mass and the Maids: power and desire in the imperial metropolis’ and chapter 3, ‘Imperial Leather: race, cross-dressing and the cult of domesticity’; Seth Koven, *Slumming: Sexual and Social Politics in Victorian London* (Princeton, 2006).

18 Matt Houlbrook, *Prince of Tricksters: the Incredible True Story of Netley Lucas, Gentleman Crook* (Chicago, 2016), 27; for discussion of accent and social boundaries, see also 37–38, 86.

19 Mrs Worthington’s daughter has ‘a loud voice/and though it’s not exactly flat/she’ll need a little more than that/to earn a living wage’: Noel Coward, *Don’t Put Your Daughter on the Stage, Mrs Worthington* (1935).

20 Rebecca Conway, ‘Making the Mill Girl Modern? Beauty, Industry, and the Popular Newspaper in 1930s’ England’, *Twentieth Century British History*, 24 (2013), 533.

21 George Orwell, *The Road to Wigan Pier* (London, 1962 [1937]), 204.

22 Richard Hoggart, *The Uses of Literacy: Aspects of Working-Class Life* (London, 2009 [1957]), 267.

23 Dave Russell, *Looking North: Northern England and the National Imagination* (Manchester, 2004), 79–110; Patrick Joyce, ‘The People’s English: Language and Class in England, *c*.1840-1920’, in Peter Burke and Roy Porter, eds, *Language, Self, and Society: a Social History of Language* (Cambridge, 1991), 154–90.

24 Hoegaerts, ‘Voices that Matter? Methods for Historians Attending to the Voices of the Past’, *Historical Reflections*, 47 (2021), 113–37.

25 Joy Damousi, *Colonial Voices: a Cultural History of English in Australia, 1840-1940* (Cambridge, 2010).

26 Hoegaerts, ‘Women’s Voices in Educational Manuals. The Gendered Sounds of Speech Therapy, Song and Education in Europe *c*.1830-1900’, *Women’s History Review*, 29 (2020), 444–64; Josephine Hoegaerts, ‘Fairness and Fluency: the Political Audibility of “newcomers” in Victorian Debating Clubs and Public Meetings, 1870-1910’, *Parliaments, Estates and Representation*, advanced access; Damousi, *Colonial Voices*, 48–75, 101–22.

27 Lynda Mugglestone, *Talking Proper: the Rise and Fall of the English Accent as a Social Symbol*, (2nd edn, Oxford, 2003 [1995]), esp. 63–69; Jurg R. Schwyter, *Dictating to the Mob: the History of the BBC Advisory Committee on Spoken English* (Oxford, 2016).

28 Joe Moran, ‘Vox populi? The Recorded Voice and Twentieth-Century British History’, *Twentieth Century British History*, 25 (2014), 463, 465–66.

29 David Cannadine, *Class in Britain* (London, 2000), 93, 147, 151.

30 Mike Savage, Fiona Devine, Niall Cunningham, Mark Taylor, Yaojun Li, Johs Hjellbrekke, Brigitte Le Roux, Sam Friedman and Andrew Miles, ‘A New Model of Social Class? Findings from the BBC’s Great British Class Survey Experiment’, *Sociology*, 47 (2013), 219–50; Mike Savage, *Identities and Social Change in Britain since 1940: a Politics of Method* (Oxford, 2010). Criticism of Savage’s analysis in the last volume, particularly of Mass Observation, has come from Florence Sutcliffe-Braithwaite, *Class, Politics and the Decline of Deference in England, 1968-2000* (Oxford, 2018), 101–22 and Jon Lawrence, *Me, Me, Me? The Search for Community in Postwar England* (Oxford, 2019). Both Lawrence and Sutcliffe-Braithwaite do, however, support readings that class identity is more complex. For more narrative and gendered readings of class see: Eve Worth, *The Welfare State Generation: Women, Agency and Class in Britain since 1945* (London, 2022) and Christina de Bellaigue, Helena Mills and Eve Worth, ‘Women, Mobility, and Education in Twentieth-Century England and Wales: a New Analytical Approach’, *Twentieth Century British History*, 33 (2022), 345–68; Stephen Brooke, ‘Gender and Working Class Identity in Britain during the 1950s’, *Journal of Social History*, 34 (2001), 773–95.

31 Hoggart, *Uses of Literacy*, 287.

32 Moran, ‘Vox Populi?’, 462.

33 Damousi, *Colonial Voices*, 3.

34 Marian Wilson Kimber, *The Elocutionists: Women, Music, and the Spoken Word* (Champaign, 2017).

35 H. St John Rumsey, *The Stammerer’s Choice: a Comparative Survey of Speech Therapy* (London, 1950), 105–6.

36 J. Raymond Solly, *Acting and the Art of Speech at the Paris Conservatoire. Hints on Reading, Reciting, Acting, and the Cure of Stammering* (Birmingham, 1891), 1–7.

37 David C.H. Wright, *The Associated Board of the Royal Schools of Music: a Social and Cultural History* (Woodbridge, 2013), 257.

38 See, for instance: Damousi, *Colonial Voices*, 151.

39 RAM Library, Syllabus of the Metropolitan Examination of the Royal Academy of Music, September 1924–April 1925.

40 Schwyter, *Dictating to the Mob*, 27.

41 Wright, *The Associated Board*, 19–41; Kimber, *The Elocutionists*.

42 Lancashire Archives, Wooler interviewed by Moore.

43 Royal College of Music Library, *The Royal College of Music, Syllabus 1906*, 9, 36.

44 RCM Library, *The Royal College of Music, Prospectus 1925-1926*, 74.

45 For the RCM, candidates still had to perform exercises in reciting poetry and prose in time to music, known as ‘attack’. RCM Library, *The Royal College of Music*, 74.

46 Private collections of the Royal Academy of Dramatic Art, *Syllabus* (1906), 10.

47 Eve Acton-Bond and Olave March, *Euchorics: the Representation of Poetry & Prosody by Means of Expressive Movement* (London, 1927).

48 According to the syllabi and prospectuses, he taught and examined at the Academy from at least 1912 to 1929.

49 RAM Library, *Syllabus of the Metropolitan Examination of the Royal Academy of Music, September 1925-April 1926*.

50 RAM Library, *Prospectus for the Royal Academy of Music, 1955-1956*.

51 This was the case for Eileen Mills, born in 1927 and profiled on the BBC Radio 4 programme *Still Lives* in 1995: British Library Sound Archive (BLSA), H5796/4, *Still Lives*, BBC Radio 4, 4 October 1995. For therapists discussing their entry into the profession, see the CST regular member *Bulletin*: e.g. Strathclyde University Library Special Collections, RCSLT/9/1/7, 1951, No. 27.

52 Jois Stansfield, ‘Giving Voice: an Oral History of Speech and Language Therapy’, *International Journal of Language and Communication Disorders*, 55 (2020), 323–24.

53 Stansfield, ‘Giving Voice’, 321.

54 Strathclyde, RCSLT/1/1, ATSD Remedial Sub-Committee Minutes, ‘Report of the meeting held at British Medical Association House on 10^th^ October 1942’. Further examples of animosity can be found in the minutes of this volume.

55 Strathclyde, RCSLT/5/1/1, CST Executive Committee Minutes: 23 November 1944, 14 December 1944; RSCLT/5/1/2, idem: 1 January 1947, 3 February 1947; RSCLT/5/1/3, idem: correspondence.

56 See: Strathclyde, RSCLT/5/1/5, Executive Committee Minutes, 1966–71.

57 Anne H. McAllister, *A Year’s Course in Speech Training* (9th edn, London, 1963 [1938]).

58 The National Archives (TNA), ED 137/399, County of Leicester Education Committee, *Annual Report of the School Medical Officer for the Year 1955*, 18–19.

59 TNA, ED 137/289, Caernarfonshire County Council, *Annual Report of the School Medical Officer for the year 1952*, 20.

60 Faulty articulation, including lisps or inability to pronounce certain consonants correctly.

61 TNA, ED 137/664, Glamorgan County Council, *Annual Report of the Principal School Medical Officer* (1959), 26.

62 Public Records Office of Northern Ireland (PRONI), LA/2/9/D/3, Armagh County Health Committee, *Annual Report of the County Medical Officer of Health for the Year 1950*, 9; LA/7/9/BF/16A, Dr Paisley to Dr Barron, 13 October 1950.

63 PRONI, LA/7/9/BF/16A, Armagh County Health Committee, ‘Speech Defects in County Armagh, N. Ireland’, September 1950, 1.

64 PRONI, LA/7/9/BF/16A, Armagh County Health Committee, ‘Speech Defects in County Armagh, N. Ireland’, September 1950, 3.

65 BLSA, H5796/4, *Still Lives*, BBC Radio 4, 4 October 1995, 13:00–15:00.

66 Stansfield, ‘Giving voice’, 323.

67
*The Teaching of English in England* (London, 1921).

68 TNA, ED 12/520, Vera Beringer to Lord Palmer, 22 March 1939.

69 TNA, ED 12/520, civil service correspondence.

70 Royal Academy of Music Library, *Prospectus 1949*, 13. The Rose Bruford College is currently located in Sidcup: <https://www.bruford.ac.uk/> accessed 9 June 2023.

71 Rose Bruford, *Speech and Drama* (London, 1948), 1. On the longer narrative of schools in promoting standard English, see Mugglestone, *Talking Proper*, 242–57.

72 Bruford, *Speech and Drama*, 29.

73 McAllister, *A Year’s Course*, 130.

74 H. St John Rumsey, *Speech Training for Children* (1st edn, London, 1937), 8.

75 Crump, *Speaking Poetry*, 196.

76 Harold J. Ripper, *Vital Speech: a Study in Perfect Utterance* (London, 1938), 15.

77 The former spelling was the opinion of the Scottish folklorist and song-collector (as well as former RAM elocution examiner) Jessie Kennedy (‘Mrs Tobias Matthay’), addressing the 1927 International Conference on Speech Training: *Proceedings of the International Conference on Speech Training held in London from 8^th^ November 1927 to 10^th^ November 1927* (London, 1928), 103. The latter was Rumsey: H. St John Rumsey, *Speech Training for Children*, 1st edn, 11.

78 Hoegaerts, ‘Stammering, Stuttering and Stumbling’, 138–41.

79 H. St John Rumsey, *Why Stammer?* (London, 1957), 10.

80 See the colophon and title pages to H. St John Rumsey, *Clear Speech: for Stage, Platform and Pulpit* (London, 1938) and Rumsey, *Why Stammer?*. It should be noted that female therapists had been undertaking such training work, without official titles, for a longer time period (particularly Elsie Fogerty at the Central School).

81 The CST had made an exemption for certain key figures, many of them male, to be examined for membership on the basis of their publications, rather than sitting the written examinations. Rumsey refused to pay the fee for this; although it may be the case that the CST introduced the fee deliberately to keep him out, committee members disagreeing fundamentally with his methods and professional attitude. I am very grateful to Jois Stansfield for this suggestion.

82 Rumsey, *Why Stammer?*, 68; Rumsey, *The Stammerer’s Choice*, vii.

83 H. St John Rumsey, *Your Speaking Voice and Its Possibilities* (London, 1936), 5.

84 H. St John Rumsey, *Speech Training for Children*, 1st edn, 25.

85 Rumsey, *Why Stammer?*, 18.

86 H. St John Rumsey, *Your Stammer and How to Correct It* (London, 1937), 64–65. He was probably referring to Elsie Fogerty’s course at the Central School.

87 Rumsey, *Speech Training for Children*, 1st edn, 76.

88 Rumsey, *Speech Training for Children* (2nd rev edn, London, 1950), 16.

89 Rumsey, *Clear Speech*, vii.

90 Rumsey, *Clear Speech*, 7–8.

91 Rumsey, *Why Stammer?*, 65.

92 Rumsey, *Clear Speech*, 9.

93 Schwyter, *Dictating to the Mob*.

94 Mugglestone, *Talking Proper*, 254.

95 Strathclyde, JCE/14/19/1/6, *Speech Training: Broadcasts to Scottish Schools Spring Term 1935*, 3.

96 Strathclyde, JCE/14/19/1/7, *Speech Training for Juniors: Broadcasts to Scottish Schools, Tuesdays 10 May-28 June 1938*, 38.

97 Strathclyde, JCE/14/19/1/6, *Speech Training: Broadcasts to Scottish Schools Summer Term 1935*, 2.

98 Strathclyde, JCE/14/19/1/7, *Speech Training for Juniors: Broadcasts to Scottish Schools, Tuesdays 10 May-28 June 1938*, 4 (emphasis in original).

99 Anne H. McAllister, *Steps in Speech Training: Step Four: Teacher’s Book* (London, 1948 [1939]), 90–93.

100 McAllister, *Steps in Speech Training*, 93.

101 McAllister, *A Year’s Course*, 130 (italics in original).

102 McAllister, *A Year’s Course*, 55.

103 McAllister, *A Year’s Course*, 55.

104 Anne H. McAllister, *The Primary Teacher’s Guide to Speech Training* (London, 1963 [1941]), 7.

105 McAllister, *The Primary Teacher’s*, 7.

106 McAllister, *A Year’s Course*, 130.

107 McAllister, *A Year’s Course*, 130–31.

108 McAllister, *The Primary Teacher’s Guide*, 2–3.

109 TNA, ED 137/30, Durham County Council, *Annual Report of the School Medical Officer* [Durham with Stockton-on-Tees as an appendix], 1950, 49.

110 Rumsey, *Speech Training for Children*, 2nd rev edn, xi.

111 Rumsey, *Speech Training for Children*, 16.

112 Rumsey, *Why Stammer?*, 22, 24.

113 Rumsey, *Your Speaking Voice*, 34; Joy Damousi, ‘“The filthy American twang”: Elocution, the Advent of American “talkies” and Australian Cultural Identity’, *The American Historical Review*, 112 (2007), 394–416.

114 H. St John Rumsey, *Speech Training: its Science and Art* (London, 1947), 2.

115 Rumsey, *Why Stammer?*, 49.

116 Nonetheless, it would take until 1974 for all aspects of speech therapy to be absorbed into NHS hospitals (from legacies in public health and the school medical services).

117 CSSD London, ‘An Interview with Voice Coach Cicely Berry by Jane Boston @CSSDLondon’, YouTube, <https://www.youtube.com/watch?v=uaLBuQW7pQg> accessed 9 June 2023.

118 Cicely Berry, *Your Voice and How to Use it Successfully* (London, 1975), 25.

119 Berry, *Your Voice and How*, 25, 49.

120 Damousi, *Colonial Voices*, 294.

121 For discussion of this through local radio, see Moran, ‘Vox Populi?’, 473; Russell, *Looking North*, 177–207.

122 Mugglestone, *Talking Proper*, 284–94.

123 RCM Library, *The Royal College of Music, Prospectus 1961-1962*, 4.

124 Personal communications from the RAM archivist, Ilse Woloszko (9, 17, and 18 May 2022) who was able to examine minute books closed to external researchers.

125 ABRSM, *Syllabus of Examinations in Speech and Drama* (London, 1951); ABRSM, *Syllabus of Speech and Drama Examinations, 1978-1979* (London, 1978); ABRSM, *Syllabus of Examinations in Speech & Drama, Acting & Communication Skills, 1986-1987* (London, 1986). Wright explains, in an appendix devoted to the ‘Speech and Drama’ examinations, which the 1986 rebrand was part of an effort to ‘broaden their appeal’ (as declining candidate enrolments meant that they were no longer cost efficient for the Board to run on a national basis): Wright, *The Associated Board*, 257–58. The LAMDA drama school is the last remaining UK provider of graded examinations in acting skills, primarily targeted to children and young adults: <https://www.lamda.ac.uk/lamda-exams/our-exams> accessed 14 June 2023.

126 Orian Brook, David O’Brien and Mark Taylor, ‘There was No Golden Age: Social Mobility into Cultural and Creative Occupations’, SocArXiv (2018), <https://doi.org/10.31235/osf.io/7njy3>.

127 John Gallagher, *Learning Languages in Early Modern England* (Oxford, 2019), 79. For the ‘extracurricular economy’ see: chapter 1, ‘Extracurricular economy: language teachers and language schools in early modern England’, 14–54.

128 Scottish Oral History Centre, University of Strathclyde, SOHC/RCSLT ‘JessW’ interviewed by Jois Stansfield, 23 March 2017, 08:27 and 37:32.

129 Personal communication from Jois Stansfield, 4 July 2023, detailing uncoded and not-yet-pseudonymised transcripts. I am exceptionally grateful to Jois Stansfield for sharing these transcripts with me and alerting me to the reflections on accent contained in them.

130 Raw, *Speech Correction*, 31.

131
*Hartlepool Northern Daily Mail*, 30 April 1931, 5; ‘Conjurer’s Action. Fellow-Magician Sued’, *Lancashire Evening Post*, 1 May 1931, 7; ‘Conjurers at Law’, *Sheffield Daily Telegraph*, 2 May 1931, 5.

